# Inflammasome Activity in Response to Influenza Vaccination Is Maintained in Monocyte-Derived Peripheral Blood Macrophages in Older Adults

**DOI:** 10.3389/fragi.2021.719103

**Published:** 2021-08-20

**Authors:** Stephen N. Crooke, Krista M. Goergen, Inna G. Ovsyannikova, Richard B. Kennedy

**Affiliations:** ^1^ Mayo Clinic Vaccine Research Group, Mayo Clinic, Rochester, MN, United States; ^2^ Department of Quantitative Health Sciences, Mayo Clinic, Rochester, MN, United States

**Keywords:** inflammasome, influenza, NLRP3, vaccination, aging

## Abstract

**Introduction:** Each year, a disproportionate number of the total seasonal influenza-related hospitalizations (90%) and deaths (70%) occur among adults who are >65 years old. Inflammasome activation has been shown to be important for protection against influenza infection in animal models but has not yet been demonstrated in humans. We hypothesized that age-related dysfunction (immunosenescence) of the inflammasome may be associated with poor influenza-vaccine response among older adults.

**Methods:** A cohort of younger (18–40 years of age) and older (≥65 years of age) adults was recruited prior to the 2014–2015 influenza season. We measured hemagglutination inhibition (HAI) titers in serum before and 28 days after receipt of the seasonal inactivated influenza vaccine. Inflammasome-related gene expression and protein secretion were quantified in monocyte-derived macrophages following stimulation with influenza A/H1N1 virus.

**Results:** Younger adults exhibited higher HAI titers compared to older adults following vaccination, although inflammasome-related protein secretion in response to influenza stimulation was similar between the age groups. Expression of *P2RX7* following influenza stimulation was lower among older adults. Interestingly, *CFLAR* expression was significantly higher among females (*p* = 2.42 × 10^−5^) following influenza stimulation and this gene may play an important role in the development of higher HAI antibody titers among older females.

**Conclusion:** Inflammasome activation in response to influenza vaccination appears to be maintained in monocyte-derived macrophages from older adults and does not explain the poor influenza vaccine responses generally observed among this age group.

## Introduction

Seasonal influenza A has varying yearly mortality and is estimated to cause 3,000–49,000 deaths annually, resulting in over 250,000 excess hospitalizations and related healthcare costs exceeding $90 billion in the US alone (Poland, 2009; Centers for Disease Control and Prevention, 2010). The 2017–2018 influenza season saw marked increases in morbidity and mortality, with 79,400 reported deaths and 959,000 influenza-related hospitalizations (Rolfes et al., 2019). According to the Centers for Disease Control and Prevention (CDC), approximately 90% of influenza-related deaths and 70% of reported hospitalizations occurred among adults ≥65 years old (Rolfes et al., 2019). Older adults suffer disproportionately from influenza—and its exacerbation of underlying co-morbidities—as a result of immunosenescence, which is a collective of inherent age-associated changes in immune cell biology that results in weakened B cell and T cell immune responses (Crooke et al., 2019a; Crooke et al., 2019b). Immunosenescence not only limits the immune response against natural infections but also compromises responses to vaccination, thus hindering the primary preventive strategy employed against seasonal influenza. Although vaccine formulations have been specifically designed to improve immune responses in older adults, influenza-specific antibody titers induced by these vaccines are often still lower than those observed in younger adults receiving a standard dose trivalent influenza vaccine (TIV) (Goodwin et al., 2006; Chen et al., 2011).

Immunosenescence is marked by a series of complex biological changes that clearly impact adaptive immunity; however, age-related changes to the innate immune system are far less understood or characterized. A limited number of studies investigating this phenomenon in the context of influenza have implicated dysregulated cytokine production as one of the primary factors associated with poor immune outcomes. Sridharan and others have observed decreased secretion of IFN type I and IFN type III from aged plasmacytoid dendritic cells (pDCs) in response to influenza virus stimulation Sridharan et al. (2011), and correlations between decreased cytokine responses and influenza-specific antibody titer have also been reported (Panda et al., 2010). Significant decreases in IL-6, TNF-α, IL-12p40, and IFN-α production have been observed following Toll-like receptor (TLR) stimulation in myeloid dendritic cells (mDCs) and pDCs from older adults, identifying strong associations between dysregulated TLR function and influenza antibody response (Panda et al., 2010). While these studies highlight important aspects of innate immunity that are impacted during aging, the full extent to which immunosenescence affects innate immunological responses to influenza virus is currently unknown.

The inflammasomes are a class of multimeric complexes comprised of NOD-like receptors (NLRs) that are responsible for the enzymatic processing and maturation of certain innate cytokines (e.g., IL-1β, IL-18) (Schroder and Tschopp, 2010), and studies have found recognition of influenza A by the inflammasome complex to be essential for establishing protective adaptive immunity (Ichinohe et al., 2009). Activation of the inflammasome occurs upon recognition of intracellular pathogens or other cellular stressors through two distinct signaling events. In the case of influenza, recognition of viral RNA by TLR7 results in the NF-κB-mediated expression of inflammatory cytokine proforms (signal 1), while the influenza virus M2 protein or the PB1-F2 polymerase stimulate activation of the NLRP3 (NOD-, LRR- and pyrin domain-containing protein 3) inflammasome complex (signal 2) (Ichinohe et al., 2010; McAuley et al., 2013). Activation of the inflammasome complex leads to increased caspase-1 activity, the processing of inflammatory cytokines into their bioactive forms, and the ultimate release of inflammatory mediators via pyroptosis (Fink and Cookson, 2005).

Multiple animal studies have demonstrated the importance of the inflammasome during influenza infection. Increased mortality, massive cellular infiltration, and elevated NO production have been observed in *Il18* ^−/−^ mice infected with influenza A (Liu et al., 2004); with increased susceptibility to influenza reported for *Nlrp3* ^−/−^ and *Casp1* ^−/−^ mice (Thomas et al., 2009). Schmitz and others observed decreased neutrophil and CD4^+^ T cell recruitment, diminished inflammatory lung infiltrates, reduced mucosal and serum IgM levels, and decreased CD4^+^ T cell activation following influenza A infection in mice deficient for the IL-1β receptor (Schmitz et al., 2005). A decline in inflammasome function with age has also been observed to contribute to increased mortality in mice following influenza infection (Stout-Delgado et al., 2012). Despite increasing evidence supporting the role of inflammasomes during influenza infection in animal models, studies investigating inflammasome activity in humans are scarce.

In this work, we sought to investigate the contributions of inflammasome activation to vaccine-induced humoral immunity as well as the impact of age and immunosenescence on inflammasome function. To do this, we recruited 147 subjects to receive a standard dose of seasonal TIV balanced by age and sex. We assessed humoral immunity post-vaccination by hemagglutination inhibition (HAI) titers and evaluated inflammasome activity in macrophages derived from peripheral blood monocytes at both the gene and protein level using reverse-transcription (RT)-PCR and cytokine ELISA. To our knowledge, this is the first study to investigate the influence of age on vaccine-induced inflammasome activation in humans.

## Methods

### Subject Recruitment

The final study cohort was comprised of 147 adult subjects divided into two subgroups on the basis of age: 18–40 years of age (*n* = 75) and ≥65 years of age (*n* = 72). All subjects in the study were recruited in 2014 from Olmsted County, MN, and the surrounding area. Each subject completed a blood draw prior to receipt of the 2014–2015 standard dose TIV, at 24 h post-vaccination, and at 28 days post-vaccination. Peripheral blood mononuclear cells (PBMCs) and serum samples collected at each timepoint were processed for cryogenic storage using previously standardized protocols (Umlauf et al., 2012). All study procedures were reviewed and approved by the Mayo Clinic Institutional Review Board, and written informed consent was obtained from each study participant.

### Hemagglutination Inhibition Titers

HAI assays were performed using a standardized procedure set forth by the Human Immunology Project Consortium (Wang et al., 2008). Briefly, serum samples were treated overnight with receptor-destroying enzyme (RDE) from *Vibrio cholerae* (MilliporeSigma; Burlington, MA) and subsequently heat-inactivated for 45 min at 56°C. Serial two-fold dilutions of RDE-treated serum (25 μl; beginning at 1:10) were prepared in 1x PBS and incubated with influenza virus A/H1N1/California/7/2009 (25 μl; hemagglutination titer = 8 HA units) in a 96-well V-bottom plate for 30 min at room temperature. A 0.5% solution of turkey red blood cells (Lampire Biological Laboratories; Pipersville, PA) was subsequently added to each sample and incubated for 45 min at 4°C. Serum samples were analyzed in duplicate with a third measurement being conducted when the initial two runs differed. The HAI titer was reported as the reciprocal of the highest dilution where hemagglutination was inhibited.

### Macrophage Culture

CD14^+^ monocytes were isolated from PBMCs and cultured for 2 weeks under conditions supporting differentiation into macrophages. Briefly, CD14^+^ monocytes were isolated from whole PBMCs through negative selection using the human Monocyte Isolation Kit II (Miltenyi Biotec, Inc.; Auburn, CA). Purified CD14^+^ monocytes were incubated in serum-free Macrophage-SFM culture media (Thermo Fisher Scientific; Waltham, MA) supplemented with 20 mM HEPES, 10 ng/mL GM-CSF, 1% penicillin/streptomycin, and 1% amphotericin B, for 2 weeks at 37°C. Fresh culture media was added every 48 h. Cells were subsequently washed with 1x PBS, and non-adherent cells were discarded. Differentiated macrophages were detached using 0.25% trypsin-EDTA, harvested, and plated for all subsequent assays. Differentiation status and purity of the macrophage population was determined visually and by flow cytometry, by decreased expression of CD14 and increased expression of CD16 (Fluorochrome-labeled antibodies obtained from BD Biosciences, CA) (Akagawa et al., 2006; Jin and Kruth, 2016).

### Inflammatory Cytokine Measurements

CD14 ^+^ macrophages were isolated from differentiation cultures and 5 × 10^4^ cells added to each well of a 96-well tissue culture plate (round bottom). Cells were incubated at 37°C with media alone (unstimulated), influenza virus A/H1N1/California/7/2009 (MOI = 0.5), resiquimod (R848, 1 μg/ml; Mabtech, Inc.; Cincinnati, OH), or both influenza virus and R848. After 24 h, cell culture supernatants were collected and assayed for IL-1β, pro-IL-1β, caspase-1, and IL-18 using Quantikine® ELISA kits (R&D Systems, Inc.; Minneapolis, MN) and Human IL-18 Instant ELISA kits (Thermo Fisher Scientific; Waltham, MA) according to the respective manufacturer’s protocols. The coefficient of variation (CV) for all cytokine ELISA assays are presented in Supplementary Table S1.

### Inflammatory Gene Expression

CD14^+^ macrophages from the cytokine assays detailed above were stored at −80°C in RNAprotect Cell Reagent (Qiagen, Inc.; Valencia, CA) until further use. Cell pellets were subsequently thawed and total RNA was extracted using RNeasy Mini kits (Qiagen, Inc.; Valencia, CA) according to the manufacturer’s protocol. Isolated RNA was quantitated, normalized for concentration across all samples, and sent to the Mayo Clinic Medical Genome Facility for quality control and analysis. All RNA samples were analyzed using an inflammasome pathway-focused RT^2^ Profiler™ PCR Array (PAHS-097Z, Qiagen, Inc.; Valencia, CA).

### Statistical Analysis

Difference between age groups in demographic variables were assessed using Pearson’s chi-square test. Correlations between variables are calculated using Spearman’s rank correlation, Wilcoxon’s rank-sum test was used to test for differences between age groups and sexes for all continuous variables. Wilcoxon’s signed-rank test was used to test for differences when the data is paired (e.g. stimulated vs unstimulated values). Gene expression values (ΔCt) were first normalized by subtracting the cycle threshold (Ct) from the corresponding *B2M* housekeeping gene from the Ct value for each gene. Log_2_ fold-changes for stimulated vs unstimulated samples were calculated by taking the difference between the ΔCt values for the unstimulated minus the ΔCt from the stimulated samples.

## Results

### Subject Demographics

The study cohort of 147 subjects ranged from 18.1 to 92.3 years of age and was designed to equally represent both younger (18–40 years of age, *n* = 75) and older (≥65 years of age, *n* = 72) age groups. The study cohort was predominantly White (89%), with the remaining individuals identifying as either Asian (3%), Other (1%), or electing not to disclose their ethnicity (7%). Most of the study participants (89%) identified as non-Hispanic or Latino. The younger and older age subgroups were 58.7 and 59.7% female, respectively. See Table 1 for a complete summary of subject demographics.

**TABLE 1:** Study cohort demographics.

	Young (N = 75)	Old (N = 72)	Total (N = 147)	*p* value
Age				
Mean (SD)	26.3 (4.03)	73.5 (5.46)	49.5 (24.1)	—
Q1, Q3	24.1, 29.1	69.3, 76.6	26.8, 72.8	—
Range	18.1–39.6	65.2–92.3	18.1–92.3	—
Sex	—	—	—	0.896^1^
Female	44 (58.7%)	43 (59.7%)	87 (59.2%)	—
Male	31 (41.3%)	29 (40.3%)	60 (40.8%)	—
Race	—	—	—	0.019^1^
White	62 (82.7%)	69 (95.8%)	131 (89.1%)	—
Asian	5 (6.7%)	0 (0%)	5 (3.4%)	—
Other	0 (0%)	1 (1.39%)	1 (0.68%)	—
Unknown	8 (10.7%)	2 (2.78%)	10 (6.8%)	—
Ethnicity	—	—	—	—
Choose Not to Disclose	0 (0%)	1 (1.39%)	1 (0.68%)	—
Hispanic or Latino	0 (0%)	1 (1.39%)	1 (0.68%)	—
Not Hispanic or Latino	64 (85.3%)	67 (93.1%)	131 (89.1%)	—
Unknown	11 (14.7%)	3 (4.17%)	14 (9.52%)	0.086^1^

1 Pearson’s Chi-squared test.

### Humoral Response to Influenza Vaccination

**FIGURE 1:**
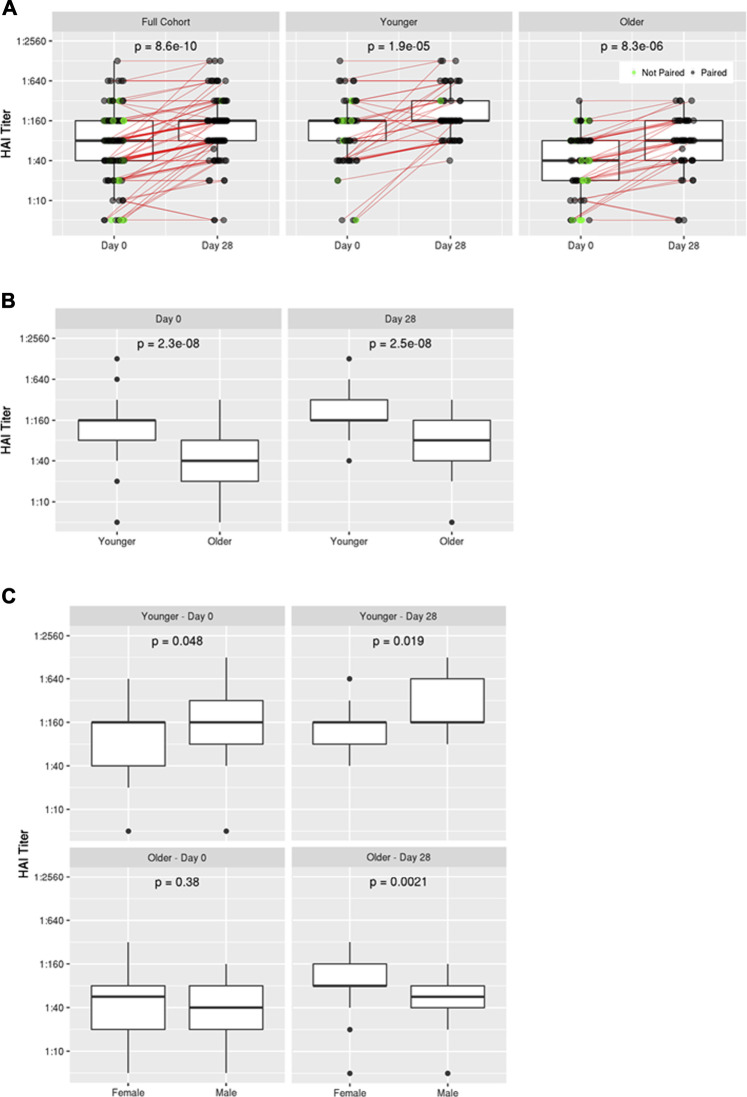
Influenza A/H1N1 HAI titers pre- and post-vaccination. **(A)** Comparison of HAI titers at Baseline (Day 0) and Day 28 for the full cohort as well as younger and older subgroups, **(B)** Comparison of HAI titers between younger and older subgroups at Day 0 and Day 28, **(C)** Comparison of HAI titers in younger and older subgroups at Day 0 and Day 28 on the basis of biological sex.

As expected, HAI titers against influenza A/H1N1 were significantly higher 28 days after vaccination with TIV (Day 28 median HAI titer = 1:160; *p* = 8.6 × 10^−10^) compared to pre-vaccination titers (Baseline median HAI titer = 1:80) in all subjects as well as within both younger (*p* = 1.9 × 10^−5^) and older (*p* = 8.3 × 10^−6^) age subgroups (Figure 1A). Younger subjects exhibited significantly higher median HAI titers compared to older subjects both at Baseline (1:160 vs 1:40; *p* = 2.3 × 10^−8^) and Day 28 (1:160 vs 1:80; *p* = 2.5 × 10^−8^) (Figure 1B). Following vaccination, 7 of 114 (6%) of the subjects were found to seroconvert (as defined by a 4-fold increase in HAI titer), and 94% of the subjects were seropositive, defined as an HAI titer ≥1:40, at Day 28 which is also considered a correlate of protection (Cox, 2013). We compared seroconversion rates in subjects with low HAI titers (<40) before vaccination to subjects with higher HAI titers (≥40) and found: 4/22 (18.2%) versus 3/92 (3.3%%) seroconverted, respectively. When comparing the younger (18–40) and older (≥65) subgroups, we found that 10% of the younger subjects seroconverted and 100% were seropositive at Day 28; while 2% of the older subjects seroconverted and 87% were seropositive at Day 28. Recognizing that the rates of seroconversion were low, we relaxed the definition to a 2-fold rise in HAI titer and repeated the analysis, revealing a similar seroconversion trend with respect to baseline HAI titer (40.9% of low HAI subjects seroconverted compared to 10.0% of high HAI subjects), and a similar rate of seroconversion for younger and older subjects (16.7% for both).

No significant difference in HAI titer was detected between men and women in the overall cohort; however, sex-based differences were apparent within each age subgroup (Figure 1C; Supplementary Figure S1). Younger men exhibited slightly higher HAI titers than young women at Baseline (*p* = 0.048), and this disparity became more apparent following vaccination (*p* = 0.019). Conversely, women in the older age group exhibited markedly higher HAI titers than men following vaccination (*p* = 0.0021), despite having similar HAI titers at Baseline (*p* = 0.38).

### Inflammasome-Mediated Protein Production Following Influenza Vaccination

In order to assess inflammasome activity, we measured IL-1β, pro-IL-1β, IL-18, and caspase-1 production by PBMCs in response to *in vitro* stimulation with the following: A/H1N1 influenza virus, resiquimod (R848, a TLR7 agonist), or a combination of A/H1N1 and R848. Stimulation of whole PBMCs resulted in high but variable levels of pro-IL-1β and IL-1β, while production of IL-18 and caspase-1 was much lower but also inconsistent (data not shown). Because monocytes and macrophages have been suggested to play a key role in immune responses to influenza with age (Roberts, 2020), we refocused our analyses on CD14^+^ peripheral blood monocytes. Monocytes were isolated using negative selection to minimize manipulation of the cells and cultured *in vitro* under conditions to promote macrophage differentiation prior to stimulation (Jin and Kruth, 2016). As expected, differentiated macrophages had an oval, “fried egg morphology” and expressed high levels of CD16 with little to no CD14 (Akagawa et al., 2006).

**FIGURE 2:**
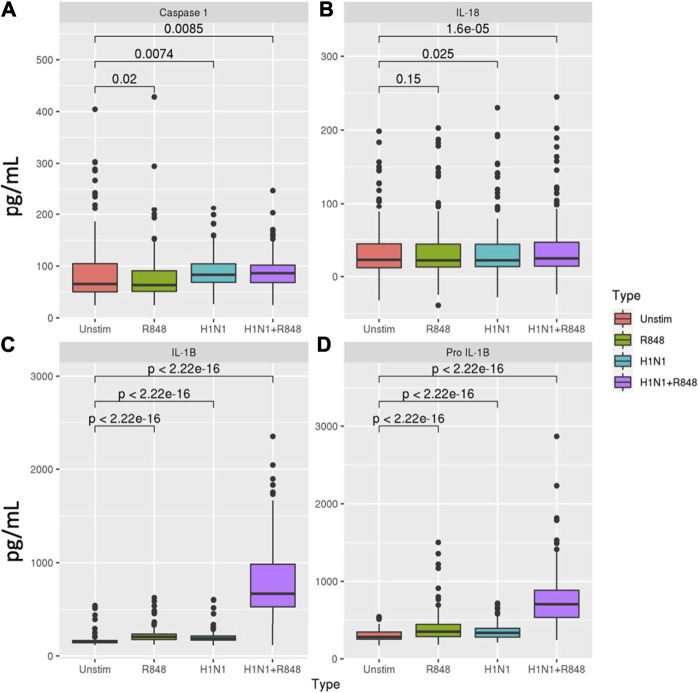
Inflammasome-related caspase and cytokine secretion profiles in macrophages. Comparison of **(A)** caspase-1, **(B)** IL-18, **(C)** IL-1β, and **(D)** pro-IL-1β secretion in response to either R848 (a TLR7 agonist), influenza A/H1N1 virus, or a combination of both stimuli for subjects with sufficient cells for evaluation (*n* = 138). *p*-values derived from Wilcoxon’s signed-rank test.

We first evaluated the extent of inflammasome activity in macrophages at baseline. Levels of inflammasome-related proteins and cytokines are summarized in Figure 2 and Supplementary Tables S2–S4. Influenza stimulation resulted in a significant percent increase in caspase-1 (27.2%, *p* = 0.007), pro-IL-1β (19.1%, *p* = 2.22 × 10^−16^), and IL-1β (22.8%, *p* = 2.22 × 10^−16^) secretion relative to unstimulated macrophages. Treatment with R848 induced similar increases in pro-IL-1β (24.5%, *p* = 2.22 × 10^−16^) and IL-1β (33.6%, *p* = 2.22 × 10^−16^) production, but a slight decrease in caspase-1 levels (-3.1%, *p* = 0.02). Combined stimulation with influenza and R848 resulted in markedly increased production of pro-IL-1β (153.9%, *p* = 2.22 × 10^−16^) and IL-1β (339.8%, *p* = 2.22 × 10^−16^), with more moderate but significant increases in caspase-1 (32.0%, *p* = 0.009) and IL-18 (8.5%, p = 2 × 10^−5^).

We next evaluated if age or biological sex were associated with baseline inflammasome activity (Supplementary Tables S3 and S4). Interestingly, only IL-1β secretion in response to influenza virus at day 1 (33.8 vs 22.4 pg/ml; *p* = 0.025) and IL-18 secretion in response to R848 at baseline (1.35 vs 0.37 pg/ml; *p* = 0.021) were significantly associated with age and both were higher in older adults. Regarding sex-based differences, females exhibited significantly higher caspase-1 (21.16 vs 3.16 pg/ml; *p* = 0.029) and pro-IL-1β levels (50.97 vs 32.9 pg/ml; *p* = 0.016) in response to influenza stimulation at baseline, whereas males secreted higher levels of pro-IL-1β (49.39 vs 24.9 pg/ml; *p* = 0.034) and IL-18 (3.06 vs 0.83 pg/ml; *p* = 0.0093) at Day 1 in response to stimulation with R848 or influenza, respectively.

Influenza vaccination did not appear to have an immediate effect on macrophage inflammasome activity, although IL-1β levels were slightly elevated in unstimulated macrophages 24 h post-vaccination compared to baseline (161.27 vs 152.01 pg/ml, *p* = 0.01). Stimulation of Day 1 macrophages with influenza virus elicited a significant increase in caspase-1 production (35.7%, *p* = 9.8 × 10^−5^), but more moderate increases in IL-18 (4.2%, *p* = 0.0013), IL-1β (15.7%, *p* = 2.22 × 10^−16^), and pro-IL-1β (13.4%, *p* = 3.43 × 10^−14^) compared to unstimulated. Treatment with R848 induced moderate increases in IL-1β (36.7%, *p* = 2.22 × 10^−16^) and pro-IL-1β (15.9%, *p* = 4.49 × 10^−15^) production, while combined influenza and R848 stimulation resulted in significant increases for all four proteins (Figure 2, Supplementary Table S2). Interestingly, the magnitude of caspase-1 (*p* = 0.012) and IL-18 (*p* = 0.024) production in response to influenza + R848 was greater at Day 1 compared to baseline, while the opposite was true for IL-1β (*p* = 0.031) and pro-IL-1β (*p* = 0.024) secretion in response to influenza alone. We did not observe any associations between inflammasome-mediated cytokine production and change in HAI titer after vaccination (R ranged from -0.057 to 0.031 and *p* > 0.56 for all 4 outcomes). Correlations with inflammasome activity were also weak to non-existent for baseline and Day 28 HAI titer.While we believe that signaling *via* TLR7 plays an important role (as evidenced by our data from cells stimulated with R848 alone), but we do not think a TLR7-related mechanism/pathway solely explains our observations. Stimulation of cells with A/H1N1 alone often resulted in similar levels of cytokine production as R848 alone (see Supplementary Table S2). Only when both signals were provided to the cells did we note a synergistic effect on cytokine/protein production–which is presumably due to complete inflammasome activation.

### Expression of Inflammasome-Related Genes Following Influenza Vaccination

**FIGURE 3:**
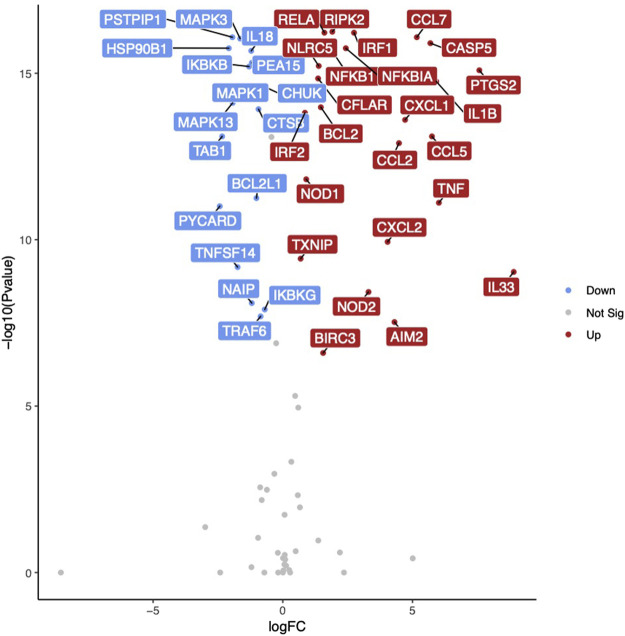
Inflammasome-related gene expression in response to influenza stimulation. Volcano plot illustrating differentially expressed genes in monocyte-derived macrophages at Baseline in response to influenza A/H1N1 + R848 stimulation. Gene names highlighted for 40 genes with lowest *p*-values. Red indicates increased gene expression, blue indicates decreased gene expression.

To further characterize the contributions of inflammasome activation to vaccine-induced influenza immunity, we isolated RNA from cultured macrophages at Baseline and evaluated the expression levels of 84 inflammasome-related genes using a custom RT-PCR array. Baseline unstimulated gene expression was associated with age in a single gene (*P2RX7*) which had a significantly lower expression in older subjects (*p* = 0.006). Comparing baseline unstimulated gene expression between sexes, eight genes (i.e., *CFLAR, NFKBIB, NFKBIA, CCL7, CHUK, HSP90AB1, RIPK2, PYCARD*) were identified with significantly higher expression levels (*p* < 0.01) among women. There were fewer differences observed between old and young individuals, suggesting that macrophages from both age groups had a similar ability to activate inflammasome pathways upon stimulation. Older subjects had greater expression of *CASP5* (*p* = 0.013) and lower expression of *CCL2* (0.043) and *CFLAR* (0.006). Following stimulation of baseline macrophages with A/H1N1 and resiquimod, we identified 51 genes with significantly altered expression levels (28 upregulated, 23 downregulated; *p* < 0.01) (Figure 3, Table 2). When evaluating the effects of both age and sex on gene expression in response to influenza stimulation, only *CFLAR* expression was statistically significant for age (*p* = 0.006), but not with sex (*p* = 0.057).

**TABLE 2:** Inflammasome-related gene expression in response to influenza stimulation.

Genes	N	Median log_2_ FC	Median FC^a^	*p*-value^b^
*RIPK2*	93	1.909	3.755	5.66E-17
*IRF1*	93	2.746	6.709	6.04E-17
*RELA*	93	1.604	3.041	6.04E-17
*CCL7*	92	5.166	35.91	8.28E-17
*PSTPIP1*	92	−1.942	0.26	8.28E-17
*MAPK3*	92	−1.642	0.32	9.14E-17
*NFKB1*	91	1.691	3.23	1.21E-16
*CASP5*	91	5.69	51.634	1.25E-16
*HSP90B1*	90	−2.079	0.237	1.77E-16
*NFKBIA*	90	2.429	5.386	1.77E-16
*IL18*	93	−1.217	0.43	2.12E-16
*PEA15*	90	−1.19	0.438	4.83E-16
*NLRC5*	89	1.385	2.611	6.04E-16
*IKBKB*	90	−1.3	0.406	6.29E-16
*PTGS2*	86	7.576	190.837	8.13E-16
*CFLAR*	90	1.361	2.568	1.43E-15
*IL1B*	86	5.916	60.379	1.76E-15
*CHUK*	93	−0.714	0.609	2.53E-15
*MAPK1*	92	−1.184	0.44	2.82E-15
*MAPK13*	82	−1.933	0.262	7.78E-15
*BCL2*	90	1.467	2.764	1.05E-14
*CTSB*	89	−0.935	0.523	1.20E-14
*IRF2*	91	0.854	1.808	1.54E-14
*CXCL1*	77	4.715	26.258	2.51E-14
*CCL5*	74	5.75	53.814	7.89E-14
*TAB1*	74	−2.337	0.198	7.89E-14
*HSP90AB1*	91	−0.437	0.739	8.23E-14
*CCL2*	73	4.479	22.294	1.26E-13
*NOD1*	88	0.903	1.87	1.53E-12
*BCL2L1*	90	−1.015	0.495	5.64E-12
*TNF*	62	6.016	64.724	7.77E-12
*PYCARD*	62	−2.43	0.186	9.91E-12
*CXCL2*	34	4.04	16.451	1.16E-10
*TXNIP*	86	0.687	1.61	3.72E-10
*TNFSF14*	74	−1.741	0.299	6.67E-10
*IL33*	31	8.903	478.796	9.31E-10
*NOD2*	29	3.305	9.886	3.73E-09
*NAIP*	45	−1.194	0.437	8.03E-09
*IKBKG*	76	−0.695	0.618	1.25E-08
*TRAF6*	77	−0.854	0.553	2.02E-08
*AIM2*	26	4.305	19.767	2.98E-08
*HSP90AA1*	93	−0.255	0.838	1.29E-07
*BIRC3*	36	1.555	2.938	2.53E-07
*PPC*	93	0.477	1.392	4.94E-06
*RTC*	90	0.596	1.512	1.11E-05
*CASP1*	90	0.329	1.256	0.000471
*P2RX7*	86	−0.324	0.799	0.00108
*MAPK9*	29	−0.871	0.547	0.00276
*SUGT1*	58	−0.613	0.654	0.00326
*PANX1*	18	0.579	1.494	0.00475
*MAP3K7*	35	−0.811	0.57	0.00665
*FADD*	17	0.66	1.58	0.011
*CASP8*	92	0.071	1.05	0.0184
*TNFSF4*	18	−2.99	0.127	0.0432
*CARD6*	14	−0.952	0.544	0.0906
*NLRP3*	7	1.368	2.582	0.109
*BIRC2*	18	0.498	1.413	0.229
*IL6*	3	2.201	4.599	0.25
*MAPK8*	76	−0.189	0.877	0.256
*NFKBIB*	89	0.076	1.054	0.295
*TAB2*	77	0.01	1.007	0.369
*CIITA*	4	5.008	55.928	0.375
*TIRAP*	24	0.094	1.067	0.406
*IRAK1*	36	0.064	1.05	0.571
*XIAP*	19	0.128	1.093	0.623
*IL12A*	6	−1.208	0.862	0.688
*NLRC4*	6	0.247	1.187	0.844
*MYD88*	89	0.023	1.016	0.867
*CARD18*	1	−2.414	0.188	1
*IFNG*	1	−8.555	0.003	1
*MAPK11*	1	−0.71	0.611	1
*NLRP1*	1	2.358	5.128	1
*NLRP9*	1	−0.179	0.883	1
*NLRX1*	1	0.283	1.216	1
*RAGE*	1	0.005	1.003	1
*CD40LG*	0	—	—	—
*IFNB1*	0	—	—	—
*IL12B*	0	—	—	—
*MAPK12*	0	—	—	—
*MEFV*	0	—	—	—
*NLRP12*	0	—	—	—
*NLRP4*	0	—	—	—
*NLRP5*	0	—	—	—
*NLRP6*	0	—	—	—
*PYDC1*	0	—	—	—
*TNFSF11*	0	—	—	—

a Represents the fold-change in ∆∆CT values for Baseline samples stimulated with influenza A/H1N1+R848.

b *p* < 0.01 was considered statistically significant. *p*-values derived from Wilcoxon’s signed-rank test.

We detected several correlations between changes in gene expression at baseline and the secretion of inflammasome-related proteins. Caspase-1 secretion at baseline was moderately associated with *TXNIP* expression (*r* = 0.278, *p* = 0.01), while IL-18 secretion at Day 1 was positively associated with *CASP1* expression (*r* = 0.344*, p* = 0.002) and negatively associated with *TNF* expression (*r* = −0.372, *p* = 0.007). Secreted levels of pro-IL-1β were positively associated with the expression of several inflammasome-related genes at baseline (*PTGS2*: *r* = 0.421, *p* < 0.005; *RELA*: *r* = 0.318, *p* = 0.002; *RIPK2*: *r* = 0.265, *p* = 0.01) and 24 h post-vaccination (*CCL2*: *r* = 0.385, *p* = 0.002; *HSP90AB1*: *r* = 0.311, *p* = 0.006). IL-1β secretion at baseline was positively associated with *CXCL1*(*r* = 0.437, *p* < 0.005) and *PTGS2* (*r* = 0.423, *p* < 0.005) expression but negatively associated with *NAIP* (*r* = -0.423, *p* = 0.004), *PEA15* (*r* = -0.297*, p* = 0.004), and *MAPK3* expression (*r* = −0.272, *p* = 0.009).

Interestingly, we did not detect any robust correlations between the expression of inflammasome-related genes in response to influenza stimulation at baseline and HAI titers at either timepoint.

## Discussion

In the current study, we evaluated the effect of age on inflammasome activation in response to the influenza vaccine among a cohort comprised equally of younger (*n* = 75) and older (*n* = 72) healthy adults. We observed lower than expected seroconversion rates in our cohort (≥4 fold rise in HAI titer). This may have been due to the considerable pre-existing immunity present in the cohort, as 75% of the cohort had titers of 1:40 at baseline. We and others have previously observed a negative correlation baseline HAI titer and seroconversion following another dose of influenza vaccine in older adults Ehrlich et al. (2012), Jacobson et al. (2015), as well as in children and adolescents (Ehrlich et al., 2012). As expected, younger adults exhibited higher median HAI titers than older adults both before and 28 days following influenza vaccination, although the majority of subjects (94%) achieved HAI titers ≥1:40 (the presumptive correlate of protection for influenza) (Cox, 2013). This is consistent with studies by our group and others that have consistently observed lower antibody responses among older adults following influenza vaccination (Hobson et al., 1972). Notably, inflammasome activation in monocyte-derived macrophages was similar between both age groups, as assessed by the production of inflammasome-associated proteins in response to influenza stimulation. In fact, IL-1β secretion in response to influenza stimulation at 24 h post-vaccination was higher in older adults. These findings provide evidence that inflammasome function in humans is not impaired with age (at least in peripheral monocytes), suggesting that other biological mechanisms are responsible for the decline in influenza vaccine response among older adults. An alternate explanation is that macrophages from older adults have diminished inflammasome activity but are more susceptible to influenza infection and the greater number of infected macrophages in our assay compensated for the reduced activity. This is an interesting possibility that is currently under investigation. Another possibility is that the 2-weeks *in vitro* culture to differentiate monocytes into macrophages obscured age-related differential inflammasome activation that might have been seen directly *ex vivo*. If this is true, our results would then indicate that monocytes derived from young and old individuals have similar differentiation capabilities.

While the expression of several genes was correlated with inflammasome-related cytokine production, we did not pursue this analysis further as there were few significant differences in the secreted levels of cytokines and proteins between age groups in our cohort. Notably, *P2XR7* gene expression was significantly lower in monocyte derived macrophages from older adults compared to those from younger adults following influenza stimulation. *P2XR7* encodes the purinergic receptor P2X7, which senses extracellular concentrations of adenosine triphosphate and has been identified as one of the major drivers of inflammasome activation (Perregaux and Gabel, 1998; Perregaux et al., 2000; Ferrari et al., 2006). Although lower expression of *P2XR7* would be expected to result in decreased inflammasome activation, we observed no such decline in the production of inflammasome-associated protein markers. This suggests that other factors compensate for the decreased expression of *P2XR7* or that an alternative mechanism unaffected by age is responsible for stimulating inflammasome activation following influenza vaccination.

We were also interested in evaluating sex-based differences in inflammasome activation and antibody response following vaccination. We and others have previously reported that females exhibit more robust humoral immune responses following vaccination (Kennedy et al., 2009; Lorenzo et al., 2011; Fink et al., 2018; Fink and Klein, 2018; Voigt et al., 2019). While this was true among the older age group, younger men exhibited significantly higher HAI titers compared to younger women following receipt of the influenza vaccine. The reasons for this sex-based disparity in HAI titer between age groups in our cohort are unclear. The cohort was a convenience sample recruited from the local population and it is possible that a sex-based recruitment bias among the younger half of the cohort occurred (e.g., differential rates of vaccination in the young men vs the young women).

Several genes associated with NF-κB regulation (e.g., *NFKBIB*, *NFKBIA*, *CHUK*, *RIPK2*) were more highly expressed among females compared to males for the entire cohort, suggestive of a greater degree of immune cell activation. Notably, *CFLAR* expression was significantly higher among females (*p* = 2.42 × 10^−5^) following influenza stimulation. The *CFLAR* gene encodes a caspase-8 regulatory protein that has been shown to play a role in governing the activation of the NLRP3 inflammasome and alternative mechanisms for inflammatory cytokine production (Wu et al., 2014). Interestingly, a model evaluating the effects of both age and sex on gene expression found that *CFLAR* was negatively associated with age (*p* = 0.006) and no longer exhibited a significant association with biological sex (*p* = 0.057). While this indicates that age has a more significant impact on *CFLAR* expression, collectively this data suggests that *CFLAR* may play a significant role in the development of higher HAI antibody titers among older females. Females have been reported to have more robust humoral immune responses to a number of vaccines, therefore these results may represent a potential mechanism for those findings. Further studies are warranted to assess the functional role of *CFLAR* in inflammasome activation with respect to both age and biological sex.

The strengths of our study include the relatively large cohort that was well-balanced and stratified with respect to both age and sex - allowing for analyses with both of these variables, the clear chronologic separation between young and old, the uniform assay measurements using established protocols, and the ability to evaluate both inflammasome-related gene expression and cytokine/caspase secretion. The limitations of our study include that the study cohort was predominantly Caucasian (89.1%), and we therefore lacked sufficient statistical power to evaluate any biological associations with race (Table 1). This may also limit the applicability of our results, as the demographics of our cohort do not reflect those of the general population. While the cohort was powered for analysis of age or sex, the group sizes became smaller when stratified by age and sex. We were also limited by the need to isolate monocytes and further differentiate them into macrophages for our measurements of inflammasome activation. We found that direct stimulation of PBMCs *ex vivo* produced very little evidence of inflammasome activity, prompting us to isolate and differentiate the monocytes. However, this *ex vivo* manipulation of monocytes may have introduced into our data artifacts that confounded the measurable effects of aging on the inflammasome activity of cells stimulated directly *ex vivo*. Nevertheless, to our knowledge, this is the first study to investigate the effect of age on inflammasome activation following influenza vaccination in humans and represents a key step toward understanding the age-associated decline in influenza vaccine response.

While the inflammasome has been implicated as a critical component for protective immunity against influenza in several animal studies, there have been no prior studies investigating this phenomenon or how it relates to vaccine response in human populations. The study reported here has extended our understanding of age-associated dysregulation of the immune response to influenza vaccination by comparing inflammasome function in older vs younger adults. Importantly, we provide evidence that macrophages from both older and younger adults are equally capable of inflammasome activation. We also demonstrate that the inflammasome response in macrophages after influenza vaccination is not significantly altered with age in humans; however, further study of this phenomenon is warranted. Additional human studies are desirable to validate our observations in this study, including a direct investigation of inflammasome activation in peripheral monocytes without differentiation into macrophages, and an examination of inflammasome function/activation in additional cell types (e.g., dendritic cells). Our results also suggest that sex-based and age-based differences in *CFLAR* expression may play a role in influenza vaccine response, and replication of this finding with further studies are warranted in order to understand the complex influence of biological sex and age on the immune response.

## Data Availability

The original contributions presented in the study are included in the article/Supplementary Material, further inquiries can be directed to the corresponding author.
