# Supplementary material for: Inflammasome Activity in Response to Influenza Vaccination Is Maintained in Monocyte-Derived Peripheral Blood Macrophages in Older Adults
Source: Front Aging. 2021 Aug 20;2:719103. doi: 10.3389/fragi.2021.719103 (PMC9261430; doi:10.3389/fragi.2021.719103)
Supplement: Supplementary file 1 [file DataSheet1.docx]

**
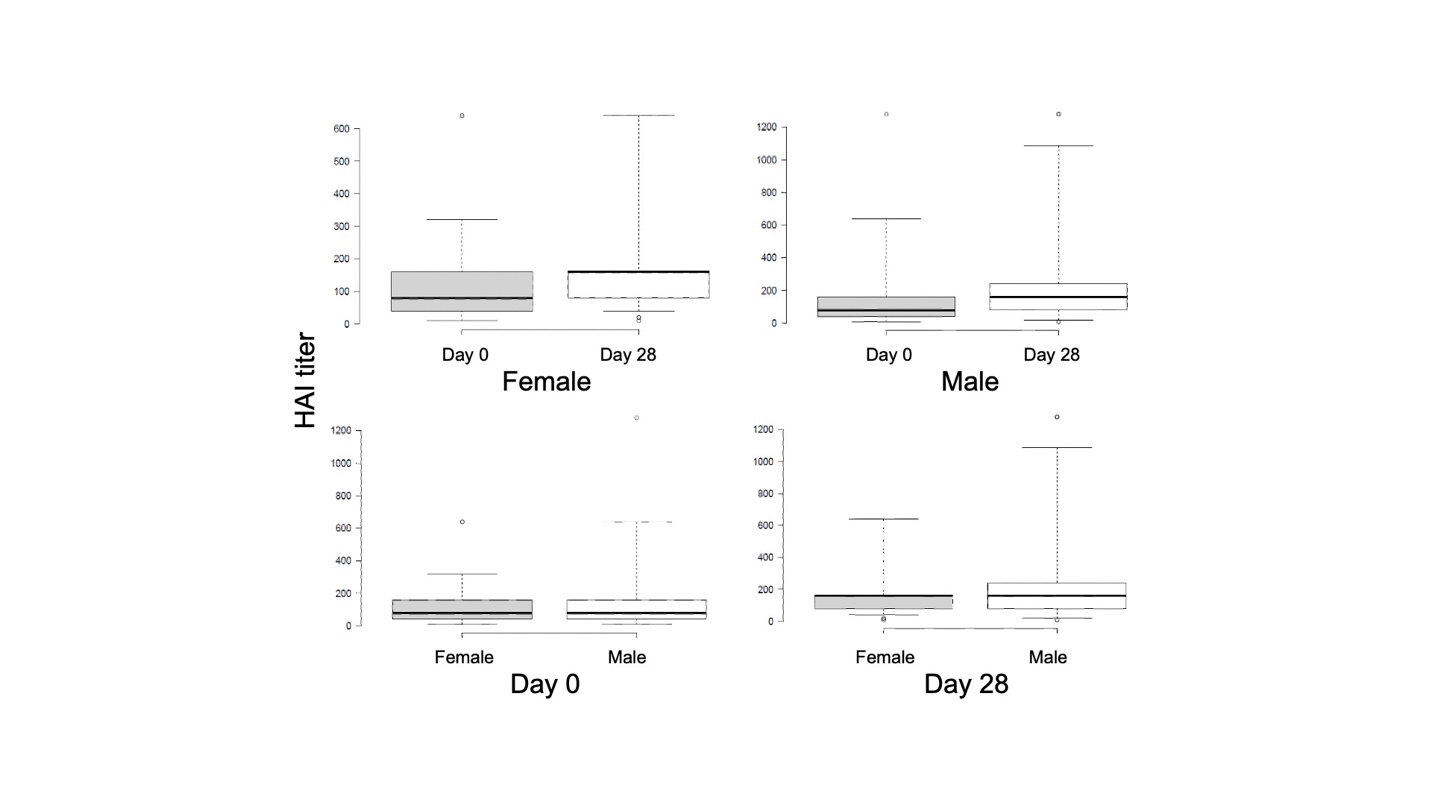
**

**Figure S1. Influenza A/H1N1 HAI titers stratified by biological sex.** Comparisons of HAI titers been male and female subgroups at Baseline (Day 0) and Day 28 for the entire cohort.

**Supplementary Table S1. Coefficients of variation for cytokine ELISA assays.**

|  | **Unstimulated** | **R848** | **H1N1** | **H1N1+R848** |
| --- | --- | --- | --- | --- |
| **Caspase-1** | 19.11 | 15.15 | 7.19 | 7.54 |
| **Pro-IL-1β** | 6.26 | 11.20 | 5.52 | 9.71 |
| **IL-1β** | 6.22 | 8.45 | 5.98 | 10.12 |
| **IL-18** | 13.84 | 22.65 | 22.26 | 18.84 |

**Supplementary Table S2. Inflammasome-related protein and cytokine secretion profiles.**

| **Analyte** | **Timepoint** | **Stimulation** | **N** | **Median (pg/mL)** | **IQR**  **(pg/mL)** | **% Change^#^** | **P-Value^#^** |
| --- | --- | --- | --- | --- | --- | --- | --- |
| Caspase 1 | Day 0 | Unstim | 138 | 65.34 | 50.14, 104.6 |  |  |
| Caspase 1 | Day 0 | R848 | 138 | 63.3 | 50.99, 90.92 | -3.12 | 0.0205 |
| Caspase 1 | Day 0 | H1N1 | 138 | 83.14 | 68.58, 104.28 | 27.24 | 0.00741 |
| Caspase 1 | Day 0 | H1N1+R848 | 138 | 86.27 | 68.19, 101.78 | 32.03 | 0.00851 |
| IL-18 | Day 0 | Unstim | 138 | 22.73 | 11.92, 44.41 |  |  |
| IL-18 | Day 0 | R848 | 138 | 22.01 | 12.96, 44 | -3.17 | 0.149 |
| IL-18 | Day 0 | H1N1 | 138 | 22.01 | 13.52, 43.82 | -3.17 | 0.025 |
| IL-18 | Day 0 | H1N1+R848 | 138 | 24.66 | 14.12, 46.56 | 8.49 | 0.000016 |
| IL-1B | Day 0 | Unstim | 138 | 152.01 | 139.92, 163.91 |  |  |
| IL-1B | Day 0 | R848 | 138 | 203.08 | 174.12, 232.75 | 33.6 | 2.22E-16 |
| IL-1B | Day 0 | H1N1 | 138 | 186.63 | 168.43, 212.1 | 22.77 | 2.22E-16 |
| IL-1B | Day 0 | H1N1+R848 | 138 | 668.51 | 528.15, 983.72 | 339.78 | 2.22E-16 |
| Pro IL-1B | Day 0 | Unstim | 138 | 278.12 | 250.17, 342.28 |  |  |
| Pro IL-1B | Day 0 | R848 | 138 | 346.21 | 283.19, 441.41 | 24.48 | 2.22E-16 |
| Pro IL-1B | Day 0 | H1N1 | 138 | 331.18 | 276.62, 389.24 | 19.08 | 2.22E-16 |
| Pro IL-1B | Day 0 | H1N1+R848 | 138 | 706 | 536.2, 885.49 | 153.85 | 2.22E-16 |
|  | | | | | | | |
| Caspase 1 | Day 1 | Unstim | 116 | 65.29 | 51.64, 98.36 |  |  |
| Caspase 1 | Day 1 | R848 | 115 | 65.14 | 50.92, 86.14 | -0.23 | 0.343 |
| Caspase 1 | Day 1 | H1N1 | 115 | 88.62 | 66.97, 105.88 | 35.73 | 0.0000984 |
| Caspase 1 | Day 1 | H1N1+R848 | 116 | 91.56 | 70.55, 115.06 | 40.24 | 0.00000773 |
| IL-18 | Day 1 | Unstim | 116 | 26.59 | 14.97, 45.47 |  |  |
| IL-18 | Day 1 | R848 | 115 | 24.94 | 14.52, 44.34 | -6.21 | 0.188 |
| IL-18 | Day 1 | H1N1 | 115 | 27.71 | 16.79, 47.81 | 4.21 | 0.00129 |
| IL-18 | Day 1 | H1N1+R848 | 116 | 29.48 | 17.27, 48.71 | 10.87 | 2.33E-08 |
| IL-1B | Day 1 | Unstim | 115 | 161.27 | 144.57, 177.31 |  |  |
| IL-1B | Day 1 | R848 | 114 | 220.44 | 186.07, 267.68 | 36.69 | 2.22E-16 |
| IL-1B | Day 1 | H1N1 | 114 | 186.61 | 168.63, 224.13 | 15.71 | 2.22E-16 |
| IL-1B | Day 1 | H1N1+R848 | 115 | 633.31 | 459.65, 1078.95 | 292.7 | 2.22E-16 |
| Pro IL-1B | Day 1 | Unstim | 116 | 292.09 | 258.69, 330.18 |  |  |
| Pro IL-1B | Day 1 | R848 | 115 | 338.63 | 299.45, 393.83 | 15.93 | 4.49E-15 |
| Pro IL-1B | Day 1 | H1N1 | 115 | 331.28 | 299.72, 362.51 | 13.42 | 3.43E-14 |
| Pro IL-1B | Day 1 | H1N1+R848 | 116 | 643.52 | 472.99, 886.4 | 120.32 | 2.22E-16 |

**^#^**Comparison to unstimulated conditions at the same timepoint.

**Supplementary Table S3. Inflammasome-related protein and cytokine secretion profiles stratified by biological sex.**

| **Analyte** | **Timepoint** | **Stimulation^#^** | **N Female** | **Median (IQR) Female** | **N Male** | **Median (IQR) Male** | **P-Value^1^** |
| --- | --- | --- | --- | --- | --- | --- | --- |
| Caspase 1 | Day 0 | R848 | 81 | 0.76 (-8.71, 6.47) | 57 | -3.32 (-29.82, 3.8) | 0.0541 |
| Caspase 1 | Day 0 | H1N1 | 81 | 21.16 (1.94, 32.21) | 57 | 3.16 (-43.66, 29.98) | 0.0287 |
| Caspase 1 | Day 0 | H1N1+R848 | 81 | 18.04 (-0.22, 30.85) | 57 | 10.91 (-41.29, 31.15) | 0.0549 |
| Pro IL-1B | Day 0 | R848 | 81 | 29.24 (4.92, 86.43) | 57 | 43.82 (9.68, 142.24) | 0.105 |
| Pro IL-1B | Day 0 | H1N1 | 81 | 50.97 (21.74, 82.76) | 57 | 32.9 (10.62, 62.76) | 0.0156 |
| Pro IL-1B | Day 0 | H1N1+R848 | 81 | 398.13 (274.66, 659.42) | 57 | 420.31 (232.97, 501.65) | 0.318 |
| IL-1B | Day 0 | R848 | 81 | 36.86 (18.44, 65.83) | 57 | 56.28 (24.21, 75.99) | 0.0951 |
| IL-1B | Day 0 | H1N1 | 81 | 28.27 (18.22, 52.54) | 57 | 28.89 (18.18, 47.89) | 0.637 |
| IL-1B | Day 0 | H1N1+R848 | 81 | 509.78 (368.79, 728.15) | 57 | 520.73 (336.35, 895.57) | 0.393 |
| IL-18 | Day 0 | R848 | 81 | 0.69 (-3.38, 4.3) | 57 | 0.61 (-1.81, 2.61) | 0.72 |
| IL-18 | Day 0 | H1N1 | 81 | 1.58 (-4.34, 5.81) | 57 | 1.72 (-0.82, 4.92) | 0.962 |
| IL-18 | Day 0 | H1N1+R848 | 81 | 3.29 (-0.86, 7.63) | 57 | 1.49 (-0.66, 4.4) | 0.137 |
| Caspase 1 | Day 1 | R848 | 66 | 0.05 (-7.33, 3.67) | 49 | 1.78 (-5.52, 5.05) | 0.338 |
| Caspase 1 | Day 1 | H1N1 | 66 | 19.73 (-3.44, 29.32) | 49 | 14.17 (-0.38, 34.29) | 0.806 |
| Caspase 1 | Day 1 | H1N1+R848 | 67 | 23.37 (-5.25, 38.88) | 49 | 14.1 (0.04, 44.91) | 0.947 |
| Pro IL-1B | Day 1 | R848 | 66 | 24.9 (3.4, 70.06) | 49 | 49.39 (16.92, 99.01) | 0.0344 |
| Pro IL-1B | Day 1 | H1N1 | 66 | 27.94 (2.32, 65.26) | 49 | 40.66 (4.21, 67.01) | 0.415 |
| Pro IL-1B | Day 1 | H1N1+R848 | 67 | 342.19 (175.29, 544.76) | 49 | 358.47 (214.85, 653.17) | 0.821 |
| IL-1B | Day 1 | R848 | 66 | 49.29 (22.58, 72.78) | 48 | 68.2 (30.89, 102.17) | 0.0938 |
| IL-1B | Day 1 | H1N1 | 66 | 26.31 (15.68, 47.29) | 48 | 26.98 (9.59, 39.78) | 0.354 |
| IL-1B | Day 1 | H1N1+R848 | 66 | 478.88 (307.95, 905.02) | 49 | 456.65 (245.5, 892.86) | 0.597 |
| IL-18 | Day 1 | R848 | 66 | 0.28 (-3.47, 3.48) | 49 | 1.09 (-1.9, 4.16) | 0.274 |
| IL-18 | Day 1 | H1N1 | 66 | 0.83 (-3.17, 5.01) | 49 | 3.06 (0.26, 7.6) | 0.00928 |
| IL-18 | Day 1 | H1N1+R848 | 67 | 2.81 (-0.63, 8.96) | 49 | 3.58 (1.35, 7.37) | 0.32 |

^#^Background corrected by subtracting unstimulated levels.

^1^ p-value calculated using Wilcoxon Rank Sum test

**Supplementary Table S4. Inflammasome-related protein and cytokine secretion profiles stratified by age.**

| **Analyte** | **Timepoint** | **Stimulation^#^** | **N Young** | **Median (IQR) Young** | **N Old** | **Median (IQR) Old** | **P-Value^1^** |
| --- | --- | --- | --- | --- | --- | --- | --- |
| Caspase 1 | Day 0 | R848 | 69 | -1.33 (-11.03, 4.2) | 69 | -2.54 (-15.98, 5.93) | 0.62 |
| Caspase 1 | Day 0 | H1N1 | 69 | 21.01 (-3.18, 33.57) | 69 | 13.22 (-19.68, 28.06) | 0.137 |
| Caspase 1 | Day 0 | H1N1+R848 | 69 | 16.6 (-1.65, 31.15) | 69 | 15.38 (-15.73, 30.53) | 0.32 |
| Pro IL-1B | Day 0 | R848 | 69 | 29.24 (5.46, 87.12) | 69 | 40.63 (5.97, 116.19) | 0.26 |
| Pro IL-1B | Day 0 | H1N1 | 69 | 42.99 (15.34, 71.06) | 69 | 42.02 (17.83, 71.94) | 0.951 |
| Pro IL-1B | Day 0 | H1N1+R848 | 69 | 363.46 (236.75, 519.88) | 69 | 440.1 (274.66, 641) | 0.108 |
| IL-1B | Day 0 | R848 | 69 | 36.86 (18.44, 71.84) | 69 | 49.98 (22.02, 79.45) | 0.375 |
| IL-1B | Day 0 | H1N1 | 69 | 28.27 (19.87, 48.48) | 69 | 28.64 (17.1, 52.54) | 0.795 |
| IL-1B | Day 0 | H1N1+R848 | 69 | 518.39 (359.47, 820.78) | 69 | 509.78 (368.79, 794.73) | 0.797 |
| IL-18 | Day 0 | R848 | 69 | 0.37 (-3.81, 2.61) | 69 | 1.35 (-1.55, 4.7) | 0.0214 |
| IL-18 | Day 0 | H1N1 | 69 | 1.38 (-4.53, 5.09) | 69 | 2.22 (-1.3, 5.15) | 0.223 |
| IL-18 | Day 0 | H1N1+R848 | 69 | 1.89 (-1.27, 6.39) | 69 | 3.14 (-0.6, 6.37) | 0.472 |
| Caspase 1 | Day 1 | R848 | 57 | 0.49 (-9.98, 5.26) | 58 | 0.11 (-5.33, 3.93) | 0.722 |
| Caspase 1 | Day 1 | H1N1 | 57 | 16.34 (-1.44, 32.19) | 58 | 16.52 (-4.28, 30.24) | 0.71 |
| Caspase 1 | Day 1 | H1N1+R848 | 57 | 24.29 (-3.6, 46.64) | 59 | 17.08 (-3.2, 34.73) | 0.566 |
| Pro IL-1B | Day 1 | R848 | 57 | 43.1 (7.15, 99.01) | 58 | 26.44 (6.5, 60.58) | 0.356 |
| Pro IL-1B | Day 1 | H1N1 | 57 | 40.39 (6.39, 67.01) | 58 | 27.76 (-0.73, 55.96) | 0.317 |
| Pro IL-1B | Day 1 | H1N1+R848 | 57 | 409.83 (232.23, 653.9) | 59 | 336.6 (137.15, 498.04) | 0.165 |
| IL-1B | Day 1 | R848 | 56 | 45.13 (16.73, 86.9) | 58 | 57.02 (34.92, 92.09) | 0.174 |
| IL-1B | Day 1 | H1N1 | 56 | 22.35 (10.34, 32.8) | 58 | 33.78 (14.78, 59.5) | 0.0246 |
| IL-1B | Day 1 | H1N1+R848 | 56 | 478.88 (289.12, 742.65) | 59 | 447.35 (297.76, 940.37) | 0.904 |
| IL-18 | Day 1 | R848 | 57 | 1.12 (-2.93, 4.86) | 58 | 0.47 (-2.81, 2.86) | 0.638 |
| IL-18 | Day 1 | H1N1 | 57 | 1.29 (-3.49, 5.07) | 58 | 2.16 (-1.19, 6.69) | 0.298 |
| IL-18 | Day 1 | H1N1+R848 | 57 | 3.03 (-0.64, 11.79) | 59 | 3.52 (0.25, 7.66) | 0.916 |

^#^Background corrected by subtracting unstimulated levels.

^1^ p-value calculated using Wilcoxon Rank Sum test
